# Effects of different exercise interventions on heart rate variability and cardiovascular health factors in older adults: a systematic review

**DOI:** 10.1186/s11556-021-00278-6

**Published:** 2021-11-17

**Authors:** Bernhard Grässler, Beatrice Thielmann, Irina Böckelmann, Anita Hökelmann

**Affiliations:** 1 Department of Sport Science, Faculty of Humanities, Otto von Guericke University, 39104 Magdeburg, Germany; 2 Department of Occupational Medicine, Faculty of Medicine, Otto von Guericke University, Magdeburg, Germany

**Keywords:** Heart rate variability, Autonomic nervous system, Exercise intervention, Cardiovascular health factors, Healthy adults, Older adults

## Abstract

**Background:**

Aging impairs physiological processes in the autonomic nervous, endocrine, and cardiovascular systems which are associated with increased risk of cardiovascular disease. Heart rate variability (HRV), the beat-to-beat variations of successive heartbeats, is an indicator of cardiac autonomic control and cardiovascular health. Physical activity has beneficial effects on cardiovascular health. However, no review has been conducted to summarize the effects of different exercise modalities on HRV in older adults. Therefore, the aim of this systematic review was to summarize the effects of endurance, resistance, coordinative, and multimodal exercise interventions on resting HRV and secondary health factors in healthy older adults aged 60 years in average and over.

**Methods:**

Five databases (PubMed, Scopus, SPORTDiscus, Ovid, and Cochrane Library) were searched for eligible studies published between 2005 and September 8th, 2020. Two reviewers independently assessed the studies for potential inclusion. Outcome measures were changes in resting HRV indices, baroreflex sensitivity, blood pressure, body fat, body mass, body mass index, cardiac output, distance in the six-minute walking test, stroke volume, total peripheral resistance, and VO_2_ max or VO_2_ peak from pre to post intervention. The methodological quality of the final data set was assessed using two scales (TESTEX and STARD_HRV_). This review was registered in PROSPERO: CRD42020206606.

**Results:**

The literature search retrieved 3991 articles, of which 13 were included in the review. Five studies used multimodal, three studies endurance, two studies resistance, two studies coordinative, and one study used an endurance and a resistance training intervention. The majority of the studies revealed significant positive effects on cardiac autonomic control, except for the resistance training interventions. All exercise modalities improved secondary health factors. The methodological quality assessment revealed a few criteria to improve the quality of and comparability between studies.

**Conclusion:**

This systematic review revealed beneficial effects on cardiac autonomic control in healthy older adults through endurance, coordinative, and multimodal training but not through resistance training. Secondary health factors improved after all types of physical interventions. Future investigations should more thoroughly adhere to methodological standards of exercise interventions and ECG recording for the assessment of autonomic regulation.

**Supplementary Information:**

The online version contains supplementary material available at 10.1186/s11556-021-00278-6.

## Introduction

People aged 65 or over make up the world’s fastest growing age group. In 2019, approximately 9% belong to this age group [1]. In particular Western societies have experienced a demographic expansion of older adults’ population due to improved medical care and declining birth rates in recent decades. For example, the proportion of people aged 65 or over in Europe and North America is as high as 18% and could rise to 25% by 2100 [1]. As longer lives are accompanied by cardiovascular and other diseases of aging, our society is facing enormous medical challenges. As a result, the burden on the healthcare system is increasing due to a growing number of patients and a greater need for therapeutic interventions and treatment of diseases. For example, global expenditures for health care increased by 3.9% annually between 2000 and 2017, reaching US$7.8 trillion in 2017 [2]. To reduce health care costs while ensuring a high quality of life, the focus should be on prevention rather than treatment [3].

Normal aging processes cause impairment of cardiac autonomic control and manifest as reduced parasympathetic modulation of the cardiovascular system [4, 5]. This parasympathetic modulation can be indexed as heart rate variability (HRV), a non-invasive measure describing beat-to-beat variations of the time intervals between successive heart beats [6–8]. These fluctuations are the result of heart-brain interactions and dynamic non-linear autonomic nervous system (ANS) processes [9]. HRV can be described with time-domain, frequency-domain, and non-linear metrics [9]. In general, good cardiac autonomic control, indexed as relatively high HRV, is related to better mental and physical health, whereas lower values are related to reduced regulatory capacity, disturbances in the cardiovascular system, and impaired adaptability to internal and external changes [8, 10]. Reduced cardiac autonomic control is associated with a number of pathological conditions, including coronary heart disease and mortality [11–13], future functional decline [14], chronic heart failure [15], sarcopenia [16], and hypertension [17]. This contributes to increased risk for cardiovascular diseases [18–20], decreased physical fitness [21], and quality of life in older adults [22].

Age-related decline in HRV has been reported in several studies [13, 23, 24], but is also the result of lifestyle factors and not aging processes alone [25, 26]. In this context, cross-sectional studies reported a positive relationship between long-term sportive lifestyle and HRV in older adults [25–28]. Moreover, HRV has also been proven to predict longevity in very old persons better than an annual health examination [29].

Apart from changes in cardiac autonomic control, aging is also associated with a progressive impairment of cardiovascular functioning. Among the affected functions are a reduced arterial baroreflex control of heart rate and increased vascular resistance favoring hypertension [30–32]. Furthermore, resting metabolic rate [33] and daily physical activity decline as a consequence of the aging process [19]. In women, estrogen production is reduced after menopause, leading to an increase in sympathetic activity and a decrease in endothelial function. These alterations increase blood pressure and favor hypertension in older women, and may account for the higher cardiovascular mortality among older women compared to older men [34].

Despite these age-related alterations in the cardiovascular system and impairments in physical performance, sedentary behavior becomes more prevalent and participation in structured exercise programs declines with increasing age [35, 36]. Indeed, previous reviews demonstrated the positive effects of physical interventions on HRV in children [37], young adults [38], and diseased individuals [39–41], and in studies using aerobic [42, 43], resistance [44], or interval training interventions [45]. However, in the older adults, the effects of physical training on HRV are scarcer and more controversial. Type, intensity, and duration of intervention are possible reasons for these discrepancies [5]. In addition, there is limited research on the effects of combined endurance and resistance training interventions [46]. Finally, we are not aware of any review considering the effects of physical interventions on cardiac autonomic control and secondary health factors in older adults. Looking at factors determining cardiovascular health, such as blood pressure, body fat, or aerobic capacity, provides a more comprehensive view of cardiovascular health than only assessing HRV. For these reasons, there is a need for a systematic review of current studies with physical interventions focusing on the effects on cardiac autonomic control in older adults. In addition to HRV as an indicator of parasympathetic modulation, we intended to consider other physiological parameters related to cardiovascular health to gain a more comprehensive picture of the effects of physical interventions on the autonomic and cardiovascular health of older adults. These secondary outcomes were extracted from the eligible studies focusing on changes in cardiac autonomic control.

The aim of this systematic review was to summarize the existing literature on the effects of exercise interventions on cardiac autonomic control and secondary health factors in healthy adults aged 60 years in average and over. Given the above described positive influence of physical training on cardiac autonomic control and cardiovascular health, we hypothesized positive effects of any type of physical intervention on resting cardiac autonomic control and cardiovascular health.

## Methods

A systematic review, investigating the effects of different exercise interventions on resting cardiac autonomic control and secondary health factors in healthy older adults, based on the Preferred Reporting Items for Systematic Reviews and Meta-Analysis (PRISMA) statement was conducted [47]. We also performed a methodological and reporting quality assessment by using the tools TESTEX [48] and STARD_HRV_ [49].

This review follows in its structure a previous review conducted by the same authors on young adults [38]. HRV variables were chosen as indicator of cardiac autonomic control because HRV is sensitive to exercise training-induced adaptations and convenient for the investigation of the effects of exercise training on the state of the ANS [7, 50]. For that purpose, the traditional exercise activities, endurance and resistance training, were included. Additionally, coordinative training, intended to improve specific motor skills, and multimodal exercise interventions, comprising at least two types of modalities, were included as well. This procedure follows our previous review with young adults [38]. Secondary to HRV indices, some cardiovascular health factors, that were evaluated in the intervention studies, were considered in this review as well. These were the following variables: baroreflex sensitivity (BR), body fat (BF), body mass (BM), body mass index (BMI), blood pressure (BP), cardiac output (CO), stroke volume (SV), total peripheral resistance (TPR), VO_2_ max or VO_2_ peak, and distance in the six-minute walking test (6-MWT). Further, methodological and reporting quality of the eligible studies were evaluated via two quality assessment tools (TESTEX and STARD_HRV_).

### Data sources and search strategy

A comprehensive, systematic search using the databases PubMed, Scopus (Elsevir), SPORTDiscus, Ovid, and Cochrane Library was conducted. The search was restricted to manuscripts published between January 1st, 2005, and September 8th, 2020. The following terms were used: (resistance training OR resistance exercise OR strength training OR strength exercise OR aerobic training OR aerobic exercise OR physical training OR physical exercise OR multimodal training OR multimodal exercise OR coordinative training OR coordinative exercise) AND (heart rate variability OR HRV OR cardiac autonomic control OR autonomic function OR parasympathetic activity OR parasympathetic nervous system OR cardiac vagal tone OR autonomic cardiac modulation OR vagus nerve OR vagal tone OR vagal activity).

### Inclusion and exclusion criteria

The inclusion criteria for relevant studies were: (1) involving at least ten healthy participants aged 60 years in average or over without diseases relevant for HRV analysis in the training group (please see for a detailed description of diseases relevant for HRV analysis: Sammito & Böckelmann [51]); (2) physical training intervention (including the following exercise modalities: endurance, resistance, coordinative, or multimodal training) with a minimum of four weeks and eight training sessions; (3) randomized controlled trials, quasi-experimental trials, cross-over controlled trials, or controlled trials without randomization; (4) measurement of at least one HRV parameter at resting position before (pre) and after (post) the intervention through Holter ECG or chest belt; (5) studies with 24-h ECG measurement when a short-term recording segment at resting position was analyzed; (6) full-text in English or German language; and (7) human participants. The exclusion criteria consisted of: (1) studies with participants with diagnosis of dementia, mental diseases, neurological diseases, endocrine diseases (diabetes, thyroid gland disease), cardiac diseases, hypertension (systolic blood pressure ≥ 140 mmHg and/or diastolic blood pressure ≥ 90 mmHg [52]), or other health-related diseases; (2) measuring acute exercise effects or HRV during exercise; (3) single-case studies, review articles, short communications, letters with insufficient information to analyze the results, guidelines, theses, dissertations, qualitative studies, scientific conference abstracts, or studies on animals; (4) 24-h ECG recording without short-term analysis at resting position; (5) HRV assessment through recording the pulse rate manually or through photoplethysmography; and (6) studies with professional athletes. 24-h ECG recordings were excluded as they do not provide standardized conditions for interindividual comparisons. In addition, comparisons between pre- and postintervention are limited because behavior and daily activities would have to be identical at both measurements. Therefore, in this review, we restricted to standardized short-term measurements under laboratory conditions to assess cardiac autonomic control.

### Data collection and analysis

#### Selection of studies

The search was applied to each electronic database and all retrieved articles were transferred to the Citavi 6 reference manager (Swiss Academic Software, Wädenswil, Switzerland). After removing duplicates, two authors (B.G. and B.T.) independently screened all titles and abstracts for retrieving relevant articles. Further, based on the criteria for inclusion and exclusion, the full-text of each relevant article was screened by the same two authors independently. Finally, the references of the eligible articles were screened for further articles. When necessary, the opinion of a third author (I.B.) was considered at any stage of the search process.

#### Data extraction

Data of the final studies sample were extracted based on the PICOS approach [53] and conducted by two authors (B.G. and B.T.) independently. The following data were extracted: sample characteristics (sample size, age, gender), HRV protocol (method [ECG or chest belt], respiration [paced or spontaneous], position [supine, sitting, standing], sampling frequency), analyzed HRV parameters, secondary outcomes (BF, BM, BMI, BP, BR, CO, SV, TPR, VO_2_ max or peak, and 6-MWT), recording length, characteristics of intervention (type, duration of intervention, sessions per week), and control group. Regarding physical training interventions, studies were considered that utilized long-term physical exercises, performed regularly in a planned, structured, and purposive manner with the objective to improve or at least maintain individual capabilities [54]. Therefore any form of endurance (aerobic), resistance, multimodal, and coordinative exercise modalities intended to improve physical performance were considered. There were no further restrictions regarding type of exercise. Therefore, any form of endurance training (e.g., continuous or interval training) and resistance training (e.g., isometric or dynamic training) was included. Likewise, forms of coordinative training were included as long as they were described as “physical activity” that contributed to the increase in energy expenditure [54]. The extracted HRV parameters with their physiological meaning are displayed in Table 1. All extracted information of the studies is summarized in Table 2. More details on physiological background of the HRV parameters can be found in the referenced literature [6, 9, 63]. Additionally, resting heart rate (RHR) and mean RR interval (mRR) were also extracted.

**Table 1:** Description of extracted HRV parameters

Variable	Units	Description	Indicator of …
SDNN	ms	Standard deviation of all NN intervals	total variability
RMSSD	ms	Square root of the mean of the sum of the squares of differences between adjacent NN intervals	short-term variability
NN50 count		Number of pairs of adjacent NN intervals differing by more than 50 ms in the entire recording	spontaneous variability
pNN50	%	NN50 count divided by the total number of all NN intervals	spontaneous variability
LF	ms^2^	Power in low frequency range (≤ 0.04 Hz)	sympathetic and parasympathetic, but predominantly sympathetic
HF	ms^2^	Power in high frequency range (0.15–0.4 Hz)	parasympathetic
LF nu		LF power in normalised units LF/(Total Power–VLF) × 100	sympathetic and parasympathetic, but predominantly sympathetic
HF nu		HF power in normalised units HF/(Total Power–VLF) × 100	parasympathetic
LF/HF		Ratio LF [ms^2^]/HF [ms2]	sympathetic and parasympathetic
TP	ms^2^	Total performance or total spectrum; corresponds to energy density between 0.00001 to 0.4 Hz	total variability
SD1	ms	Standard deviation of instantaneous beat-to-beat variability, extracted from Poincaré Plot	parasympathetic
SD2	ms	Standard deviation of the long-term variability, extracted from Poincaré Plot	sympathetic and parasympathetic

HF (nu), power in high frequency range (in normalized units); Hz, Hertz; LF (nu), power in low frequency range (in normalized units); ms, millisecond; NN50, number of pairs of adjacent NN intervals differing by more than 50 ms in the entire recording; pNN50, number of pairs of adjacent NN intervals differing by more than 50 ms in the entire recording divided by the total number of all NN intervals; RMSSD, square root of the mean of the sum of the squares of differences between adjacent NN intervals; SD1, standard deviation of instantaneous beat-to-beat variability, extracted from Poincaré Plot; SD2, standard deviation of the long-term variability, extracted from Poincaré Plot; SDNN, standard deviation of NN intervals. Modified Sammito & Böckelmann [55].

**Table 2:** Characteristics of the studies included and TESTEX and STARD_HRV_ score

Author, year	Participants (sample size, age [year], gender)	HRV protocol (method, respiration, position, sampling frequency)	Analysis length	Intervention (type, duration, sessions/week)	Control group	TESTEX	STARD_**HRV**_
**Endurance training**							
Albinet et al., 2016 [56]	32	Polar RS800 chest belt	256 NN intervals	Endurance (aquaerobics & swimming)	yes (stretching)	10.5	19.5
	Age: 67 ± 5	n.r.		21 weeks			
	26 women, 6 men	sitting n.r.		2 sessions			
Albinet et al., 2010 [5]	24	Polar RS800 chest belt	256 NN intervals	Endurance (walking, circuit-training, stepping & running)	yes (stretching)	11	21
	Age: 70.7 ± 4.2	n.r.		12 weeks			
	13 women, 9 men	sitting n.r.		3 sessions			
Okazaki et al., 2005 [57]	10	ECG	6 min in each breathing mode	Endurance (walking, running, swimming & cycling)	no	4.5	20.5
	Age: 71.3 ± 3.0	spontaneous & paced (12 breaths/min)		12 months			
	4 women, 6 men	supine 1.000 Hz		3 sessions			
Wanderley et al., 2013 [22]e	50	PolarNV vantage chest belt	5 min	Endurance (walking, stepping, dancing)	yes	10	18.5
	Age: 68.6 ± 5.7	spontaneous		8 months			
	39 women, 11 men	supine n.r.		3 sessions			
**Resistance training**							
Gerage et al., 2013 [58]	29	Polar s810i chest belt	5 min	Resistance	yes (stretching)	12.5	22.5
	Age: 65.9 ± 4.6	spontaneous		12 weeks			
	100% women	Sitting n.r.		3 session			
Kanegusuku et al., 2015 [18]	25	ECG	5 min	High-intensity progressive resistance training	yes	9.5	23
	Age: 63.5	n.r.		16 weeks			
	18 women, 7 men	sitting 500 Hz		2 sessions			
Wanderley et al., 2013 [22]	50	PolarNV vantage chest belt	5 min	Whole-body resistance	yes	10	18.5
	Age: 68.6 ± 5.7	spontaneous		8 months			
	39 women, 11 men	supine n.r.		3 sessions			
**Coordinative training**							
Audette et al., 2006 [21]	19	ECG	5 min	Coordinative (Tai Chi)	yes	8.5	18.5
	Age: 72.3	spontaneous		12 weeks			
	100% women	n.r. n.r.		3 sessions			
Varas-Diaz et al., 2020 [20]	20	Polar RS800CX chest belt	5 min	Coordinative (dancing)	yes	10.5	19.5
	Age: 68.6 ± 5.7	n.r.		6 weeks			
	7 women, 13 men	supine 1.000 Hz		5 (week 1–2), 3 (week 3–4) & 2 sessions (week 5–6)			
**Multimodal training**							
Eggenberger et al., 2020 [59]	70	Polar RS800CX chest belt	5 min	Multimodal (DG: dancing, resistance & balance; MG: treadmill walking, memory training, resistance & balance; TG: treadmill walking, resistance & balance)	no	7	21
	Age: 78.8	n.r.		26 weeks			
	45 women, 25 men	sitting 1.000 Hz		2 sessions			
McKune et al., 2017 [46]	58	Suunto t6 chest belt	300 NN intervals	Multimodal (endurance, resistance & balance)	no	7	18.5
	Age: 71.8	n.r.		12 weeks			
	44 women, 14 men	sitting n.r.		2 or 3 sessions			
Rezende Barbosa et al., 2019 [60]	39	Polar RS 800 chest belt	1.000 NN intervals	Multimodal (resistance, coordination, balance, agility & walking)	yes	9.5	20
	Age: 59.2	n.r.		18 weeks			
	100% women	supine n.r.		3 sessions			
Rossi et al., 2013 [61]	17	Polar s810i chest belt	256 NN intervals	Multimodal (endurance & resistance)	yes	4.5	18
	Age: 60.8 ± 6.2	spontaneous		16 weeks			
	100% women	supine n.r.		3 sessions			
Verheyden et al., 2006 [62]	29	ECG	10 min	Multimodal (endurance & resistance)	yes	10.5	19,5
	Age: 63.3	spontaneous		12 months			
	100% men	standing & supine 1.000 Hz		2–3 sessions			

BF, body fat; BM, body mass; BMI, body mass index; BP, blood pressure; BR, baroreflex sensitivity; CO, cardiac output; DG, dancing group; ECG, electrocardiography; HF (nu), power in high frequency range (in normalized units); HF%, relative power in high frequency range; LF (nu), power in low frequency range (in normalized units); LF%, relative power in low frequency range; ln, natural logarithm; MG, memory training group; mRR, mean RR interval; n.r., not reported; pNN50, number of pairs of adjacent NN intervals differing by more than 50 ms in the entire recording divided by the total number of all NN intervals; RHR, resting heart rate; RMSSD, square root of the mean of the sum of the squares of differences between adjacent NN intervals; SD1, standard deviation of instantaneous beat-to-beat variability, extracted from Poincaré Plot; SD2, standard deviation of the long-term variability, extracted from Poincaré Plot; SDNN, standard deviation of NN intervals; SV, stroke volume; TG, treadmill walking group; TP, total power; TPR, total peripheral resistance; TV, total variance; VO_2_ max, maximum oxygen consumption; VO_2_ peak, peak oxygen consumption; 6-MWT, six minute walking test

#### Quality assessment

Two assessment tools were applied for evaluation of the methodological and reporting quality of the selected studies: “Tool for the Assessment of Study Quality and reporting in Exercise (TESTEX) scale” [48] and “STARD_HRV_” [49]. The first tool is designed to evaluate the quality of exercise training studies by using twelve items with a maximum score of 15 points. The second tool focuses on the quality of ECG recording, processing, and analyzing of HRV parameters. It includes 25 items and has a maximum score of 25 points. Two authors (B.G. and B.T.) evaluated the quality of the included studies independently. Again, any conflicts were resolved in consultation with a third author (I.B.). We used slightly modified versions of the tools, which were already used in a previous review [38] and are described in the additional files 1 and 2.

#### Data synthesis and analysis

The changes in HRV measures and secondary outcomes of the intervention groups from pre to post measurement of the included interventions were collected and summarized in Table 3. Upward pointing arrows indicate a significant increase, downward pointing arrows a significant decrease. Horizontal arrows indicate no change.

**Table 3:** Outcome of selected heart rate related variables and secondary health factors

Outcome heart rate related variables											
Author, year	Analysis	RHR	mRR	(ln) SDNN	(ln) RMSSD	(ln) HF (nu)	(ln) LF (nu)	LF/HF	(ln) TP	SD1	SD2
**Endurance training**											
Albinet et al., 2016 [56]	Group x session interaction				↑	↑	↔				
	Within				↑	↑	↔				
Albinet et al., 2010 [5]	Group x session interaction			↑	↑	↑	↔				
Okazaki et al., 2005 [57]	Within	↔		↑		↔	↔				
Wanderley et al., 2013 [22]	Within	↓		↔		↔					
**Resistance training**											
Gerage et al., 2013 [58]	Within	↔	↔	↔	↔	↔	↔	↔		↔	↔
Kanegusuku et al., 2015 [18]	Within	↔				↔	↔	↔			
Wanderley et al., 2013 [22]	Within	↔		↔		↔					
**Coordinative training**											
Audette et al., 2006 [21]	Within					↑	↓	↔			
Varas-Diaz et al., 2020 [20]	Within				↑	↑		↔			
**Multimodal training**											
Eggenberger et al., 2020 [59]	Within (assessed from graphics)			DG, MG & TG: ↔	DG, MG & TG: ↔	DG, MG & TG: ↔					
	Between			DG & MG vs. TG: ↑; DG vs. MG: ↑	DG & MG vs. TG: ↔; DG vs. MG: ↑	DG vs. MG: ↑					
McKune et al., 2017 [46]	Within	2TG: ↘; 3TG: ↔			2TG: ↔; 3TG: ↓	2TG: ↔; 3TG: ↓	2TG: ↔; 3TG: ↓	2TG & 3TG: ↔	2TG: ↔; 3TG: ↓		
Rezende Barbosa et al., 2019 [60]	Between	↓	↑	↔	↑	↔	↑				
Rossi et al., 2013 [61]	Within	↓	↑	↔	↑	↑	↔ (LF); ↓ (LF nu)	↓			
Verheyden et al., 2006 [62]	Within		standing & supine: ↔			standing & supine: ↔	standing & supine: ↔	Standing & supine: ↔	standing & supine: ↔		
**Outcome secondary health factors**											
**Author, year**	**Analysis**	**BF**	**BM**	**BMI**	**BP**	**BR**	**CO**	**SV**	**TPR**	**VO**_**2**_ **max or peak**	**6-MWT**
**Endurance training**											
Albinet et al., 2016 [56]	Group x session interaction									↑	
Okazaki et al., 2005 [57]	Within		↓		↓ (DBP); ↘ (SBP)	↔	↔	↑	↓	↑	
Wanderley et al., 2013 [22]	Within	↓		↔	↓						↑
**Resistance training**											
Gerage et al., 2013 [58]	Within	↔	↔	↔	↔ (DBP & MBP); ↓ (SBP)						
Kanegusuku et al., 2015 [18]	Within				↔	↔					
Wanderley et al., 2013 [22]	Within	↓		↔	↔						↔
**Coordinative training**											
Audette et al., 2006 [21]	Within									↑	
Varas-Diaz et al., 2020 [20]	Within									↑	↑
**Multimodal training**											
McKune et al., 2017 [46]	Within		2TG & 3TG: ↔	2TG & 3TG: ↔	2TG: ↓; 3TG: ↓ (DBP), ↔ (SBP)						
Rezende Barbosa et al., 2019 [60]					↓						
Verheyden et al., 2006 [62]					↔					↑	

BF, body fat; BM, body mass; BMI, body mass index; BR, baroreflex sensitivity; CO, cardiac output; DBP, diastolic blood pressure; DG, dancing group; HF (nu), power in high frequency range (in normalized units); LF (nu), power in low frequency range (in normalized units); ln, natural logarithm; MBP, mean blood pressure; MG, memory training group; mRR, mean RR interval; RHR, resting heart rate; RMSSD, square root of the mean of the sum of the squares of differences between adjacent NN intervals; SBP, systolic blood pressure; SD1, standard deviation of instantaneous beat-to-beat variability, extracted from Poincaré Plot; SD2, standard deviation of the long-term variability, extracted from Poincaré Plot; SDNN, standard deviation of NN intervals; SV, stroke volume; TG, treadmill walking group; TP, total power; TPR, total peripheral resistance; VO_2_ max, maximum oxygen consumption; VO_2_ peak, peak oxygen consumption; 6-MWT, six minute walking test; 2TG: training group exercising two times per week; 3TG: training group exercising three times per week. ↑ indicates significant increase (*p* < 0.05); ↓ indicates significant decrease; ↔ indicates no significant change

## Results

### Study selection

**Fig. 1:**
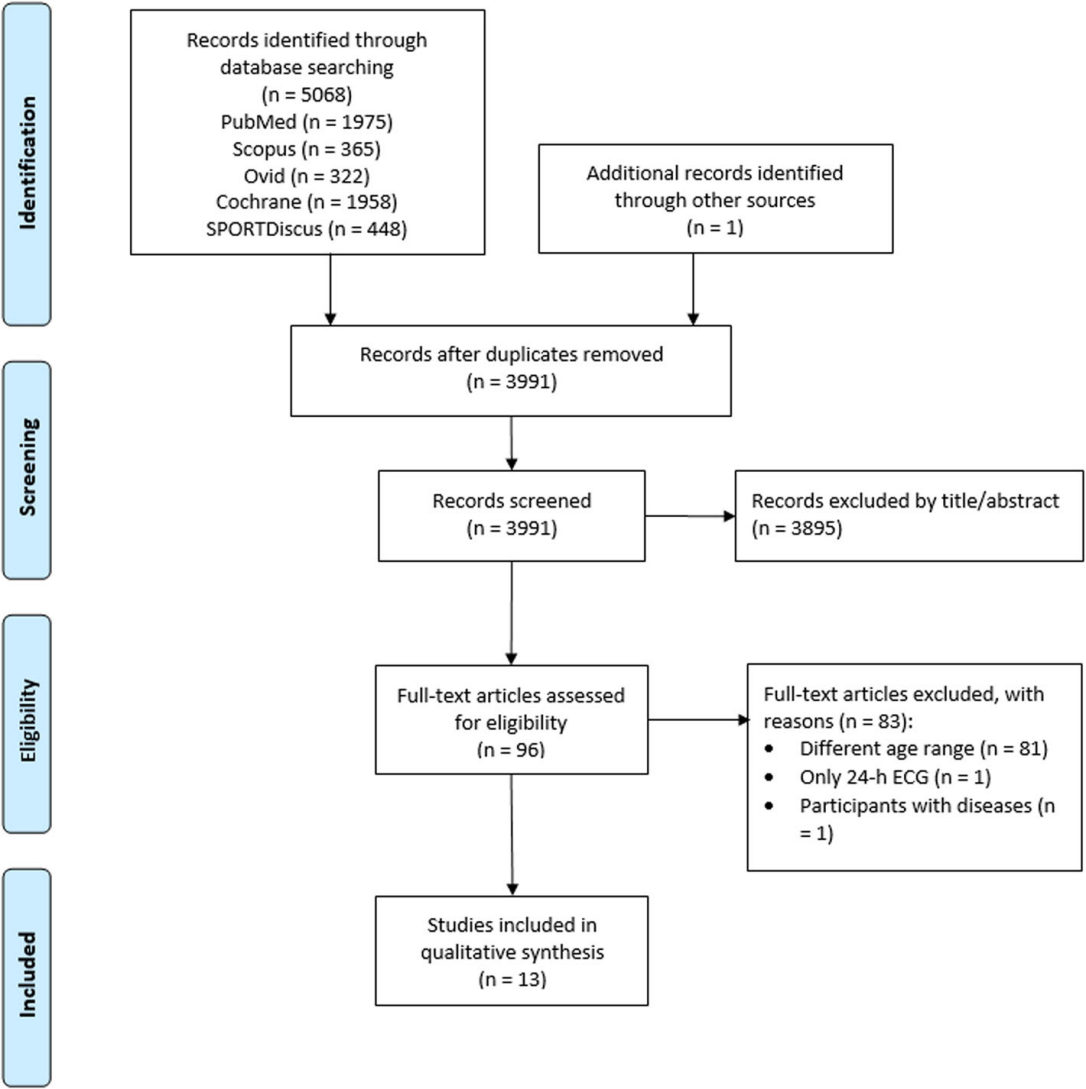
PRISMA flow diagram showing identified, included, and excluded studies

The database search initially identified 5068 records. After removing 1078 duplicates and adding one article through other sources, the titles and abstracts of 3991 articles were screened. 96 articles remained for full-text analysis. Finally, 13 studies fulfilled all inclusion and exclusion criteria. The selection process is shown in the PRISMA flow diagram in Fig. 1.

### Study characteristics

The characteristics of the 13 eligible studies and scores in TESTEX and STARD_HRV_ are outlined in Table 2. The outcome of the most frequently used heart rate related parameters and secondary outcomes can be found in Table 3. A detailed description of the characteristics of the included studies and the results of all heart rate related variables are available in the additional files 3 and 4, respectively.

#### Participants characteristics

In total, 422 participants were included, of which 289 were allocated to training groups and 133 to control groups. 300 participants were women and 122 were men. Three studies did not include a control group [46, 57, 59]. Three studies included two or three training groups [22, 46, 59]. In the study of Audette et al. [21], the effects of practicing Tai Chi and brisk walking was compared. The walking group was not included as training group in this review since the sample size was < 10. Sample sizes ranged from ten [57] to 70 participants [59]. The mean age of the collated sample was 67.8 ± 5.4 years and ranged from 59.2 [60] to 78.8 years [59]. We still included the intervention of Rezende Barbosa et al. [60] because the mean age of the training group was 60.0 years, which was one of our inclusion criteria. The mean age of the training groups of the final sample was 69.3 ± 6.00 years and the mean age of the control groups was 67.3 ± 6.3 years. Regarding training groups, the mean age ranged from 60.0 [60] to 80.7 years [59]. Regarding control groups, the mean age ranged from 58.5 [60, 61] to 78.8 years [22]. Eight trials comprised female and male participants [5, 18, 20, 22, 46, 56, 59, 60]. Four trials considered only female [21, 58, 60, 61] and one study included only male participants [62]. All participants were in a general healthy physical and mental condition without diseases affecting cardiac autonomic control [51], but they were sedentary or in an untrained physical state. In one investigation, 28% of the participants had an BMI > 30 kg/m^2^ [22].

#### HRV and secondary health factors measurement

Four of 13 studies used ECG to record the heart rate [18, 21, 57, 62]. The remaining nine studies used chest belts. Six studies reported their breathing protocol [21, 22, 57, 58, 61, 62]. While in five studies a spontaneous breathing protocol was applied [21, 22, 58, 61, 62], a spontaneous and a paced breathing protocol was applied in one study [57]. The participants` position during the ECG recording varied between the studies. One study [22] recorded in supine and standing position. Six studies recorded while the participants were sitting [5, 18, 46, 56, 58, 59]. A supine position was chosen in five studies [20, 22, 57, 60, 61] and one study did not provide any information about the position during the recording [21]. Eight studies recorded NN intervals for a fixed time range. Six studies recorded for five minutes [18, 20–22, 58, 59], one study for six minutes during both breathing modes [57], and one study recorded for ten minutes [62]. Three studies analyzed 256 NN intervals [5, 56, 61], one study 300 NN intervals [46], and one study analyzed 1000 NN intervals [60]. Only five studies provided any information about the sampling frequency. Four studies recorded ECG with 1000 Hz [20, 57, 59, 62] and one study recorded with 500 Hz [18]. All 13 studies analyzed frequency-domain parameters. All studies except one [21] analyzed time-domain parameters and only one study used non-linear parameters [58], namely SD1 and SD2. All studies used the HRV parameter HF (ms^2^, nu, ln, or %). LF (ms^2^, nu, ln, or %) was used in ten studies. Only three studies did not apply LF [20, 22, 59]. Total power was reported in two studies [46, 62]. RMSSD was the most frequently used time-domain parameter. Eight studies used it [5, 20, 46, 56, 58–61]. Seven studies analyzed SDNN [5, 22, 57–61]. pNN50 [20] and the unusual parameter total variance [18] were used in one trial each. RHR were reported in eight studies [18, 22, 46, 57, 58, 60–62] and mRR in four studies [58, 60–62].

Three studies did not report any secondary health factor [5, 59, 61]. Six studies analyzed resting BP [18, 22, 46, 58, 60, 62]. Four studies analyzed VO_2_ max or VO_2_ peak [20, 21, 56, 62] and four studies recorded BM and BMI [22, 46, 58, 62]. The 6-MWT was applied in three studies [20, 22, 46]. Only two studies measured BF [22, 58] and two studies evaluated BR [18, 57].

#### Physical training protocols

Five studies applied multimodal exercise interventions. Two of them used a combination of endurance and resistance training [61, 62]. One study [46] included balance, endurance, and resistance training. The participants in another study [60] trained their strength, coordination, balance, and agility as well as their aerobic capacity through a walking program. Three training groups exercised in the final study [59]. All groups performed strength and balance exercises. In addition, one group also performed a virtual reality dance program (DG, dance group), another group a treadmill walking program with simultaneous verbal memory training (MG, memory group), and the final group a treadmill walking program without memory training (TG, treadmill group). Endurance training only was used in four studies [5, 22, 56, 57]. One study [22] included an endurance and a resistance training group. A form of resistance training was used in two studies [18, 58]. Finally, coordinative exercises were performed by the participants in two studies. Participants in one study [20] had to dance and participants in another study [21] practiced Tai Chi. The duration of the interventions varied between six weeks [20] and one year [57, 62] and lasted in average 20.4 weeks. In all studies, participants exercised for two or three sessions per week. However, in the first two weeks of one intervention [20], participants had to dance five times per week, in the next two weeks three times per week, and in the last two weeks two times per week.

### Heart rate related variables

#### Endurance training

Four studies with types of endurance training were retrieved from the literature search. The interventions included aquaerobics and swimming [56], walking, circuit-training, stepping, and running [5], walking, running, swimming, and cycling [57], and walking, stepping, and dancing [22]. All four interventions included men and women. Within-group or between-group analysis demonstrated significant improvements following training interventions for HF and RMSSD [5, 56], SDNN [5, 57], and HF/(LF + HF) [56]. RHR significantly decreased after one intervention [22]. Interestingly, another intervention [57] showed an increase of HRV in sedentary older participants after the intervention with similar HRV values to those of master athletes and sedentary younger adults who were studied before the intervention but did not undergo the intervention.

#### Resistance training

Three studies examined the effects of whole-body resistance training on cardiac autonomic control [18, 22, 58]. One study examined only women [58] and two studies examined men and women [18, 22]. No study found significant changes in HRV, mRR, or RHR.

#### Coordinative training

Literature search retrieved two studies using coordinative exercise interventions. In one study, female participants practiced Tai Chi [21] and in the other study men and women practiced an exergaming-based dance program [20]. After the interventions, HF nu [20, 21] and RMSSD [20] improved significantly, and LF nu decreased significantly [21].

#### Multimodal training

Significant within-group or between-group increases were detected for RMSSD [59–61], HF [59], HF nu [61], and mRR [60, 61]. Significant decreases were found in RHR [60, 61], LF nu, and LF/HF [61]. In one study [59], the pre-post comparisons within groups were estimated from the graphs because no statistical significance was provided. The graphs showed a clear improvement of HF, RMSSD, and SDNN in the group performing the multimodal training with dancing as main component of the training. In the same study, the two combined cognitive-motor training groups (DG and MG) were compared with the exclusively physical training group (TG). A significant increase in SDNN for the first groups, but no change in the TG were detected. Furthermore, HF, RMSSD, and SDNN significantly increased in the DG while it remained unchanged in the TG. In contrast to the previous results, significant reductions of ln HF, ln LF, RMSSD, and total power (TP) were detected in the study of McKune et al. [46] in the training group exercising three times per week. The training group exercising only two times per week showed no significant changes in any of the HRV parameters.

### Secondary health factors

#### Endurance training

All studies except one [5] evaluated health factors secondary to HRV and found positive improvements. VO_2_ max [56, 57], 6-MWT [22], and SV [57] significantly increased, while BM, TPR [57], BF [22], and BP [22, 57] significantly decreased.

#### Resistance training

All three studies using resistance training evaluated secondary health factors [18, 22, 58]. A significant reduction of systolic BP was reported in one study [58]. BF was significantly reduced after another intervention [22].

#### Coordinative training

Secondary health factors were measured in two interventions [20, 21]. Both interventions lead to significant improvements in VO_2_ max. In addition, 6-MWT distance increased in the study of Varas-Diaz et al. [20].

#### Multimodal training

Three of five studies assessed secondary health factors [46, 60, 62]. Multimodal training interventions were able to reduce diastolic BP [46, 60] and improve VO_2_ peak [62]. Interestingly, while participants exercising two times per week significantly improved diastolic and systolic BP, participants exercising three times per week significantly improved only diastolic BP.

### Quality assessment

The methodological and reporting quality of the studies was assessed via the tools TESTEX and STARD_HRV_. The total scores are shown in Table 3. A detailed summary is displayed in the additional file 5 for TESTEX and in the additional file 6 for STARD_HRV_.

The average score of the TESTEX was 8.88 ± 2.47 and ranged from 4.5 [57, 61] to 12.5 points [58]. However, Okazaki et al. [57] did not include a control group and was therefore limited to a maximum score of eight points. Nearly all studies specified eligibility criteria in sufficient detail except one [46]. All but one study allocated their participants randomly to the groups. Participants in the control group of the study of Audette et al. [21] were not randomly allocated to the control group. No study concealed the allocation of the participants and performed an intention-to-treat analysis. In only two studies [21, 58], assessors were blinded to group allocation.

The score of the STARD_HRV_ ranged from 18 [61] to 23 points [18]. The average score was 20.00 ± 1.56 points. All studies fulfilled the items 1, 2, 14, 18, 20, and 24. Only half a point was deducted in the items 3 [22] and 9 [21]. Twelve of 13 points were awarded in the items 4, 7, and 8. Furthermore, 11.5 points were awarded in the items 5 and 19. Contrarily, only five studies calculated their intended sample size [18, 21, 58–60]. Finally, acknowledgement of breathing [18, 21, 57, 58, 61, 62], reasons for missing data, along with percentage of missing data [5, 21, 22, 46, 56, 59, 60], and artefact cleaning methods with percentage of corrected beats were further items, that were fulfilled by only a small number of studies. The last item was fulfilled in sufficient detail only in three studies [5, 18, 59].

## Discussion

### Purpose and main findings

The present systematic review was conducted to summarize the existing literature on the effects of different exercise interventions on heart rate related variables including HRV parameters, reflecting cardiac autonomic control in older adults aged 60 years in average and over. In addition, we also considered health-related secondary variables that were assessed in these studies to look for possible interactions between changes in HRV and these secondary health factors. 13 endurance, resistance, coordinative, or multimodal training interventions, lasting for at least four weeks, were considered as eligible. Literature review revealed endurance, multimodal, and forms of coordinative training as appropriate modalities to improve cardiac autonomic control and secondary health factors in sedentary, healthy older adults. Ten of the 13 studies evaluated at least one secondary health factor. All but one study [18] demonstrated a significant improvement in at least one parameter. Furthermore, we evaluated the methodological and study reporting quality of the eligible studies through two assessment tools: TESTEX and STARD_HRV_. The evaluation showed quite good quality in reporting the methods and results of the studies. A few limitations concern the processing and analysis of HRV.

### Heart rate related variables

#### Endurance training

Three of four studies applying endurance training interventions demonstrated significant improvements in HRV parameters [5, 56, 57]. No significant changes in HRV were reported in the study of Wanderley et al. [22], despite the second longest intervention period of the four endurance interventions was applied in this study and no obvious major differences were identifiable between the studies regarding their training protocols. However, the authors stated that participants with greater subclinical inflammation at baseline showed higher HRV improvements after the intervention. In fact, exercise and training variables, as well as training principles must be taken into account when planning physical training [54]. Concerning older adults, vigorous intensity of ~ 75% HRmax for ~ 200 min/week for at least 6 to 12 months are recommended to improve health state [57]. This must be countered by the fact that participants in two studies [5, 56] could increase their HRV even with lower intensities and shorter intervention periods.

RHR decreased in one study [22] and may be explained by a decreased sympathetic outflow and decreased intrinsic heart rate [64]. The positive effects on cardiac autonomic control in the remaining studies could be attributed to the individualization of the training programs and the gradual increase of the training intensities [5, 56, 57]. Interestingly, significant effects on HRV were detected only for global and parasympathetic components of HRV (SDNN, RMSSD, and HF), while LF, which reflects a mix of sympathetic and parasympathetic activity [9], did not change significantly in none of these three studies. In conclusion, the studies demonstrated positive effects on parasympathetic modulation in older adults. Endurance training seems to be equally effective in improving cardiac autonomic control in older as in younger adults [38, 65].

#### Resistance training

Regarding resistance training, literature revealed controversial effects on HRV in older adults [18]. Similarly, our systematic review did not find any positive effects of resistance training on HRV, irrespective of the training intensities applied in the interventions. One explanation for the missing HRV changes in the present review may be the dynamic nature of the resistance training since isometric training lead to positive effects on parasympathetic modulation in a previous study [66]. Furthermore, the small sample sizes in all training groups have to be mentioned as limitation. Finally, aging is supposed to reduce HRV sensitivity to training [42, 67] since studies with young participants showed beneficial effects of resistance training on HRV [38, 68]. Resistance training is supposed to decrease blood flow in the presence of increased vascular stiffness due to normal aging processes [69]. This might be the reason why resistance training interventions do not improve cardiac autonomic control in older adults. In conclusion, resistance training seems to be less effective in improving cardiac autonomic control in older adults. Given the positive effects of endurance training on HRV as stated above and in a previous review [70], it is recommended to include aerobic elements when planning exercise training programs for older adults.

#### Coordinative training

Two coordinative training interventions demonstrated beneficial effects on cardiac autonomic control [20, 21]. The beneficial effects of Tai Chi has already been demonstrated in previous studies showing acute HRV increases after a single session of Tai Chi [71, 72]. We suggest that long-term practice of Tai Chi could lead to manifest adaptations of cardiac autonomic control and increase HRV. Despite the short intervention period of six weeks in the study of Varas-Diaz et al. [20] significant improvements were produced, that might be the result of the high training frequency in the first two weeks of the intervention. The number of training sessions per week decreased from five in the first two weeks to two sessions in the last two weeks. The results indicate dancing for older adults as a suitable training modality to improve cardiac autonomic control. In addition, compared to traditional, conditioning-oriented training modalities, it is a well-tolerated aerobic exercise, it increases motivation, and there are no safety concerns [73].

In conclusion, dancing and Tai Chi are suggested as proper training modalities to improve cardiac autonomic control in older adults. However, further studies are required to investigate the required intensity and volume to stimulate the ANS since no information were given about the intensity in the interventions. Given the small number of participants in both studies, interventions with bigger sample sizes are necessary for more powerful conclusions.

#### Multimodal training

While significant improvements in cardiac autonomic control were reported [59–61], no changes [62] and even significant reductions [46] were reported after multimodal training interventions as well. The results of the study of McKune et al. [46] with two training groups are inconclusive. On the one hand, the training group exercising two times per week showed no significant changes in HRV parameters. On the other hand, the group exercising three times per week showed significant reductions in HF, LF, RMSSD, and TP [46]. This result contradicts Raffin et al. [43] recommending higher training frequency for better improvements in cardiac autonomic control. These findings could be the result of differing levels of baseline vagal activity between the training groups [42]. Participants of the study of McKune et al. [46] had to rate their perceived exertion during each exercise session to ensure that the prescribed intensity was maintained. On a scale from 0 to 10, exercise intensity was set at 2 to 4 indicating an intensity from “easy” to “sort of hard”. This relatively low intensity may be another explanation for the absence of significant improvements in this study.

Although the participants exercised two to three times per week for one year, no significant effects on cardiac autonomic control were detected in the study of Verheyden et al. [62]. This finding contradicts other study results showing positive effects with even shorter intervention periods and a similar number of training sessions per week [59–61]. However, the authors speculated that small training effects overlapped with larger interindividual differences in HRV [62]. Furthermore, physical training interventions involving low intensity resistance training (i.e., low weights and high number of repetitions) might have no effects on cardiac autonomic control [62]. Instead, other authors recommend strenuous endurance training to improve autonomic functioning [74, 75].

In conclusion, the effects of multimodal training interventions on cardiac autonomic control are somewhat vague. Although a recording length of at least five minutes is recommended for short-term measurements [63], two studies used a specific number of NN intervals, namely 256 [61] and 300 NN intervals [46]. Finally, as resistance training interventions did not show significant positive effects on resting autonomic control but endurance training interventions did, resistance training within multimodal interventions could exert negative influences on cardiac autonomic control.

### Secondary health factors

#### Endurance training

Endurance training interventions lead to significant improvements in certain secondary health factors, including VO_2_ max [56, 57], 6-MWT [22], and BP [22, 57]. Although improvements in several secondary health variables were observed in the study of Wanderley et al. [22], no improvements were observed in autonomic measures. In contrast, Albinet et al. [56] and Okazaki et al. [57] observed improvements in HRV as well as in secondary factors. Decreased arterial compliance leads to age-related impairment of cardiovagal BR [30]. Although physical activity is supposed to attenuate this process and preserve BR [76], BR was not improved, even with higher exercise intensities and remained lower than that of master athletes and young controls at baseline in the study of Okazaki et al. [57]. However, BR might be improved by a prolonged training intervention combined with other therapies designed to improve arterial stiffness. Interestingly, both groups in one intervention [56], swimming and stretching group, equally improved VO_2_ max. This unexpected finding may be the result of the pedestrian nature of the test. According to the authors, a water-based test could have resulted in stronger improvements in the swimming group. On the other hand, the lower VO_2_ max of the stretching group at baseline could have biased the results. Therefore, this group had a higher ability to improve aerobic power.

In light of the increased cardiovascular risk in older adults due to an age-related increase in vascular stiffness contributing to systolic hypertension [77], increase in cardiac sympathetic modulation [31], changes in the endocrine system [78], as well as attenuated autonomic modulation [79, 80], the beneficial effects of endurance interventions are of great importance. In summary, as it was expected, endurance training is an adequate type of intervention to reduce the cardiovascular risk in older adults.

#### Resistance training

Resistance training showed contradicting results regarding BP with no [18, 22] or positive effects [58]. The absence of BP changes are in line with the small but non-significant reductions in HRV [18, 22]. High-intensity resistance training is supposed to increase heart rate and BP acutely through increased arterial stiffness [81, 82] and sympathetic modulation [4]. Indeed, increased arterial stiffness could be harmful to the cardiovascular system [18]. Therefore, resistance training should be used carefully in older adults and the risk of hypertension should be clarified before beginning any training intervention. Furthermore, in contrast to the resistance training group, the endurance training group was able to reduce BP significantly indicating that endurance training is better able to reduce BP than resistance training [22]. Since both groups did not significantly improve HRV, it may be speculated that the two training modalities address different physiological mechanisms.

The results regarding the change in body composition are likewise inconclusive. A reduction in BF was demonstrated in one study [22] but not in another [58]. This reduction was already shown in a previous study [83]. In both studies [22, 58], BM and BMI remained unchanged. The intervention of the study of Gerage et al. [58] lasted for only 12 weeks. This may be an explanation for the absence of BF reduction compared to the study of Wanderley et al. [22] applying an intervention period of 8 months. Furthermore, Wanderley et al. [22] mentioned the high drop-out rate in the resistance training group as one limitation. These results are noteworthy in that a recent review demonstrated that resistance training has beneficial effects on body composition in older individuals [84]. However, two studies are not enough to draw final conclusions regarding change in body composition after resistance training. In conclusion, the baseline level of the participants regarding body composition, the objective of the intervention, and the training protocol play an important role in the change of body composition. For example, high-intensity resistance training with strong hypertrophic character will not be suitable to reduce BM or BMI. However, it potentially reduces BF equally well as endurance training.

#### Coordinative training

Both coordinative training interventions lead to positive effects on cardiovascular fitness [20, 21], which is in line with the HRV improvements. Dancing as proper training modality to improve aerobic capacity and cardiovascular health has been shown in previous studies [85]. Dancing is supposed to keep BP constant and therefore ensuring adequate perfusion to vital organs [20, 86, 87]. Finally, dancing has been shown to improve quality of life, psychological well-being, oxygen uptake, metabolic parameters, and positively affects brain volumes in older adults [85, 88, 89]. Therefore, dancing seems to be a physical activity affecting the physical state positively through other mechanisms, namely social aspects. Based on the promising results of the present investigations, more studies should be conducted to investigate the effects of different kinds of coordinative training modalities on various secondary health factors. Additionally, other cardiovascular factors, such as BP and BR, should be assessed in order to evaluate the effects on cardiovascular health and potentially link it to the evaluation of cardiac autonomic control. However, the training load should be monitored carefully for optimally tailoring the training program individually and comparing the effects on ardiovascular health with other training modalities.

#### Multimodal training

While BP decreased in two studies with relatively short intervention periods of twelve [46] and 18 weeks [60], participants in another study [62] showed no BP changes after one year of training, despite participants of this and another intervention [46] showed similar baseline BP levels. Given the increased BP of the training group compared to the control group [60], the decrease after the combined intervention in the training group is not surprising and supported by a further study [90]. In summary, multimodal training interventions seem to be moderately appropriate to decrease BP.

Despite no improvement in BP, aerobic capacity increased after one intervention [62]. This finding could be the consequence of the strong aerobic character of this intervention because participants had to perform seven exercises with two sets and 20 to 30 repetitions. Contrarily, McKune et al. [46] assessed aerobic capacity with the 6-MWT but did not detect significant changes. This could be the result of the low training volume and intensity, which was insufficient to induce effects on the cardiorespiratory system, as participants rated their perceived exertion and should stay at a score of 2 to 4 out of a maximum of 10.

The diverging results regarding secondary factors are in line with the HRV results showing improvements [60], no changes [62], or contradicting results [46]. In conclusion, more studies investigating secondary health factors like BP and body composition are necessary to elucidate the exact effects of multimodal interventions. Furthermore, concurrent training of two or more conditional skills requires a sophisticated interpretation of the effects. One possibility to address this issue is to have several training groups in parallel in order to study the isolated and combined effects on cardiovascular variables.

### Possible mechanisms behind autonomic and cardiovascular adaptations

Although the exact mechanisms behind the autonomic adaptations through physical activity are not completely understood and remains to be fully elucidated [91], several possible mechanisms have been identified.

Mechanisms leading to HRV improvements through aerobic training involve the decrease in plasma norepinephrine concentration [92, 93], suppression of angiotensin II expression [94], and reduction of oxidative stress by lowering the expression of nicotinamide adenine dinucleotide phosphate oxidase [95]. Those mechanisms lead to a decrease of sympathetic influences on heart rate at rest lowering resting heart rate [93, 96]. Normalization of excitatory glutamatergic and angiotensinergic mechanisms within the paraventricular nucleus also contributes to the reduction in sympathetic outflow [97]. Stimulation of nitric oxide syntheses [91, 94, 98–100] and anti-inflammatory processes after aerobic training [99, 101, 102] may be also involved in increasing cardiac autonomic control. Finally, it is also suggested that changes in body composition and enhanced lactate tolerance as a result of long-term aerobic training positively affects cardiac autonomic control [44].

Concerning resistance training, it is proposed that reductions in the concentration of metabolites and pro-inflammatory cytokines as well as the decrease in plasma concentration of norepinephrine positively influences autonomic functioning [103]. In addition, improved baroreflex sensitivity, vascular adaptations, and increased nitric oxide synthesis could contribute to improved autonomic functioning after resistance training with low [104] or moderate [105] intensities.

The effects of coordinative training on HRV depend substantially on the intensity of the intervention and are the result of the sum of acyclic movements in the coordinative program. Pal et al. [106] proposed increased levels of serotonin and dopamine, and decreased levels of epinephrine and norepinephrine as underlying reasons for improved HRV after a coordinative exercise program with yoga. Another intervention involving capoeira indicated the decreased psychological stress and anxiety as well as socialization, friendship, and relaxation as possible contributors to improved parasympathetic modulation [107].

Since previous reviews with younger populations have found stronger positive effects of physical training on HRV than this review [38, 65], it may be speculated that ageing impairs the ability to improve HRV [67]. For example, resistance training interventions did not lead to significant improvements in HRV [18, 22, 58]. This is supported by studies that found a reduced adaptive capacity of the cardiovascular system of older adults [108, 109]. One possible reason could be the loss of cardiac neural network function with ageing [110]. However, other studies showed similar improvements in young and older adults in HRV after aerobic training [57, 74, 111].

The mechanisms that contribute to the improvement of autonomic and cardiovascular function partially overlap. For example, a decrease in inflammatory markers leads to a decrease in BP and increase in HRV [22]. The improved blood flow after resistance training enhances BR [105] and decreases BP through reduced peripheral vascular resistance [112]. It has been proposed that preserved BR sensitivity in fit older adults is related to preserved mechanical transduction by an improved arterial compliance and neural transduction [30, 76]. BR adaptations might also occur as a result of high BP peaks during resistance training lowering peripheral vascular resistance and finally reduce BP after resistance training [112]. Furthermore, vasodilation in metabolically active muscles through nitric oxide and other endothelium-dependent and independent mediators is another explanation for reduced BP after resistance training [113]. Improved blood flow after endurance training also improves cardiorespiratory fitness (i.e., VO_2_ max) [114].

### Quality assessment

#### TESTEX

The methodological and reporting quality of the included studies was analyzed by using the tool TESTEX. Based on our assessment, the studies scored between 4.5 [57, 61] and 12.5 points [58]. Since one investigation [57] did not include a control group, the maximum score in this trial was limited to eight points. The mean score of all 13 studies was 8.9 ± 2.5 points. This is higher than the studies achieved in our previous review with young adults [38]. However, 8.9 out of 15 possible points means that less than 60% of the criteria were met. Two criteria, namely allocation concealment and intention-to-treat-analysis, were fulfilled by none of the studies. Additionally, in only two studies [21, 58], the assessors were blinded to group allocation and activity was sufficiently monitored in only three studies [5, 56, 58]. Despite these shortcomings, there were some points that were fully met by almost all studies: specification of eligibility criteria (12 of 13), specification of randomization (12 of 13), and keeping relative exercise intensity constant (11 of 13).

#### STARD_HRV_

The quality score of NN interval recording, processing, and analyzing assessed by the tool STARD_HRV_, ranged between 18 [61] and 23 points [18], with an average score of 20.0 ± 1.6 points. As for the TESTEX, this is a higher score than achieved in our previous review with young adults [38], where the mean was 18.44 points. Six criteria where fully met by all interventions: 1, 2, 14, 18, 20, and 24. However, a few criteria were fulfilled only by a small number of studies: intended sample size and how it was determined (5 of 13), acknowledgement of breathing (5 of 13), and artefact cleaning methods with percentage of corrected beats (3 of 13). Especially the last two criteria are crucial for a good quality of study reporting in HRV research. Therefore, we recommend to follow the prescribed criteria to improve the quality of and comparability between studies.

#### Practical implications and future directions

The literature showed beneficial effects of different kinds of exercise training on cardiac autonomic control in healthy older adults. However, the research also showed that there is still a need for some research. As most of the studies did not compare the effects on cardiac autonomic control between women and men, no gender-specific effects could be assessed. Therefore, we recommend to investigate the differences in HRV changes in women and men in more detail since the decrease in estrogen production in older women increases the cardiovascular risk of postmenopausal compared to premenopausal women [115]. This review identified only one study measuring non-linear parameters [58]. Therefore, future studies should not only consider the traditional time- and frequency-domain parameters, namely SDNN, RMSSD, and HF, but non-linear parameters should be analyzed as well. These parameters provide a different view on heart rate regulation, namely heart rate complexity, and they could be used as “a reliable marker of complications and mortality in patients with cardiovascular disease” [116]. Heart rate fragmentation could serve as an additional biomarker in the assessment of cardiovascular risk [117]. Hence, this relatively new marker could be measured as well when assessing the risk for adverse cardiovascular events in healthy adults without clinical cardiovascular disease. Furthermore, analyzing cardiac autonomic control at several time points during the intervention would provide a more accurate reflection of the effectiveness of the intervention. Exercise-induced adaptations of cardiac autonomic control can be well detected by weekly averaging the parameter RMSSD [118, 119] because single measurements reduce the sensitivity to identify changes in HRV [118]. In addition to resting state measurements, HRV measurements during the orthostatic test [120, 121] or during walking [118] are practical alternatives for evaluating cardiac autonomic control. Follow-up analysis would give an estimation of how long the effects of a physical intervention remain. Finally, we also recommend to include a passive control group and compare the effects on autonomic functioning with this group.

The quality assessment revealed some important aspects regarding ECG recording and processing, and study reporting. Based on the results of TESTEX and STARD_HRV_, we recommend to follow the criteria of these two tools. Regarding ECG measurement and HRV analysis, we recommend to follow the criteria stated in recent reviews [122, 123]. Furthermore, we recommend to state training adherence rate and ensure a minimum of 48 h between the last training session and ECG measurement to avoid acute training effects on parasympathetic modulation [124]. Regarding ECG recording, a high sampling frequency of 1000 Hz is recommended [55] to avoid errors in the recognition of the QRS complex [125].

### Strengths and limitations

This systematic review has a few limitations. First, we did not conduct a meta-analysis, which could provide a quantitative view on the effects of physical training on HRV. Instead, we performed a qualitative analysis with the main focus on providing a comprehensive summary of the effects of different exercise modalities on resting HRV and secondary health factors in healthy older adults. We decided to refrain from a quantitative analysis due to the high heterogeneity of the studies in relation to the intervention modality, exercise protocol, and HRV analysis. The literature search was limited to English and German articles. Therefore, it is possible that we missed studies in other languages. Another limitation concerns the age criterion. Since we used an average sample-based value, namely 60 years as lower limit, participants in the retrieved studies could also be under 60 years. Thus, the results are generally not applicable to people over 60 years of age. Furthermore, we included only short-term ECG measurements. 24-h ECG recordings would provide additional information about the ANS during daily activities. Future studies should also look at 24-h recordings. It should also be considered that HRV is an indirect measure of ANS activity [126, 127] and HRV improvements after exercise interventions might be due to sinus node and heart remodeling itself rather than increases of parasympathetic control [128]. Finally, it should also be mentioned that when using data editing software of heart rate monitors, the HF band of women over 60 years of age demonstrates limited agreement with traditional ECG [129]. Therefore, traditional ECG systems and manual editing of NN intervals should be used whenever possible [129].

However, this review also has several strengths. To the best of our knowledge, it is the first review summarizing the effects of different exercise modalities on cardiac autonomic control and cardiovascular health in healthy older adults. Thus, it provides a comprehensive overview of the current literature regarding physical interventions to improve autonomic control of heart rate in healthy older adults. We described the exercise protocols, NN interval recording, HRV analyzing processes, and the outcomes of the studies thoroughly. Furthermore, we analyzed the methodological and reporting quality of the studies via two assessment tools, namely TESTEX and STARD_HRV_. Finally, we described strengths and limitations of the studies, as well as possible mechanisms for HRV improvements or non-improvements with a focus on the older population.

## Conclusion

This systematic review showed that endurance, multimodal, and coordinative training interventions are appropriate modalities to improve cardiac autonomic control in healthy older adults aged 60 years in average and over, indexed as HRV. These results indicate that these exercise modalities are equally effective in older adults as in younger adults. However, isolated resistance training without including aerobic training do not significantly improve cardiac autonomic control. In this review, the effects on secondary health factors were evaluated as well, providing additional information about the effects of exercise training on cardiovascular health status. All exercise modalities contribute to the reduction of risk factors, namely a reduction in blood pressure, body fat, body mass, total peripheral resistance, and an increase in VO_2_ max, stroke volume, distance covered in the 6-MWT, and VO_2_ peak. It should be noted that these health factors were collected as secondary outcomes and considered only in studies assessing HRV as a primary outcome. Therefore, the significance of these secondary outcomes is limited. The physiological mechanisms responsible for these adaptations are not yet fully understood. Further studies are needed to reveal the underlying physiological background leading to improved parasympathetic autonomic control and reduction of the risk for adverse cardiovascular events.

Apart from analyzing the effects of the training interventions, the methodological quality of the studies was also assessed using the tools TESTEX and STARD_HRV_. The quality assessment revealed a few deficits in the reporting of the methodologies and HRV processing. For better comparability between studies, we recommend to adhere to the criteria of these two tools. Based on the qualitative analysis in this review, we recommend the combination of different exercise modalities to improve autonomic control and reduce cardiovascular risk. A certain amount of exercise intensity and volume is necessary to stimulate the ANS and cardiovascular system. Leisure-time recreational exercises seems to be insufficient to induce positive effects on cardiac autonomic control. Dancing seems to be a very good exercise modality for older adults. It combines coordinative and conditional skills and counteracts the detrimental effects on the aging brain. We also recommend to assess not only the autonomic and cardiovascular systems but also other physiological systems such as the endocrine system as it is involved in the development of frailty. When planning training interventions, programs should be individually tailored and a gradual increase in training intensity should be ensured for optimal adaptation. Finally, aerobic interventions with at least two training sessions per week has been proved to positively influence cardiac autonomic control in healthy older adults. Contrarily, resistance training, especially high-intensity resistance training, is inappropriate to improve cardiac autonomic control.

## Supplementary Information

**Additional file 1.** Description of each modified TESTEX criterion.

**Additional file 2.** Description of each modified STARD_HRV_ criterion.

**Additional file 3.** Detailed description of the characteristics of the included studies.

**Additional file 4.** Detailed results of the heart rate related variables.

**Additional file 5.** Detailed results of the TESTEX scale.

**Additional file 6.** Detailed results of the STARD_HRV_ scale.

## Data Availability

All data generated or analyzed during this study are included in this published article and its supplementary information files.

## Abbreviations

ANS: autonomic nervous system
BF: body fat
BM: body mass
BMI: body mass index
BP: blood pressure
BR: baroreflex sensitivity
CO: cardiac output
ECG: electrocardiography
HF: power in high frequency range (0.15–0.40 Hz)
HRV: heart rate variability
LF: power in high frequency range (0.04–0.14 Hz)
mRR: mean RR interval
nu: normalized units
pNN50: number of pairs of adjacent NN intervals differing by more than 50 ms in the entire recording divided by the total number of all NN intervals
RHR: resting heart rate
RMSSD: square root of the mean of the sum of the squares of differences between adjacent NN intervals
SDNN: standard deviation of all NN intervals
SD1: standard deviation of instantaneous beat-to-beat variability, extracted from Poincaré Plot
SD2: standard deviation of the long-term variability, extracted from Poincaré Plot
SV: stroke volume
TP: total power
TPR: total peripheral resistance
VO2 max: maximum oxygen consumption
VO2 peak: peak oxygen consumption
6-MWT: six-minute walking distance test
